# A LiDAR Point Cloud Data-Based Method for Evaluating Strain on a Curved Steel Plate Subjected to Lateral Pressure

**DOI:** 10.3390/s20030721

**Published:** 2020-01-28

**Authors:** Hyeon Cheol Jo, Hong-Gyoo Sohn, Yun Mook Lim

**Affiliations:** Department of Civil and Environmental Engineering, Yonsei University, 50, Yonsei-ro, Seodaemoon-gu, Seoul 03722, Korea; brown1988@yonsei.ac.kr (H.C.J.); Sohn1@yonsei.ac.kr (H.-G.S.)

**Keywords:** LiDAR system, 3D point cloud data, structural health monitoring, safety assessment, steel plate, interpolation method, strain evaluation method

## Abstract

Structural health monitoring (SHM) and safety assessment are very important areas for evaluating the behavior of structures. Various wired and wireless sensors can measure the physical responses of structures, such as displacement or strain. One recently developed wireless technique is a light imaging detection and ranging (LiDAR) system that can remotely acquire three-dimensional (3D) high-precision coordinate information using 3D laser scanning. LiDAR systems have been previously used in geographic information systems (GIS) to collect information on geography and terrain. Recently, however, LiDAR is used in the SHM field to analyze structural behavior, as it can remotely detect the surface and deformation shape of structures without the need for attached sensors. This study demonstrates a strain evaluation method using a LiDAR system in order to analyze the behavior of steel structures. To evaluate the strains of structures from the initial and deformed shape, a combination of distributed 3D point cloud data and finite element methods (FEM) was used. The distributed 3D point cloud data were reconstructed into a 3D mesh model, and strains were calculated using the FEM. By using the proposed method, the strain could be calculated at any point on a structure for SHM and safety assessment during construction.

## 1. Introduction

In recent years, as structures have become increasingly diversified in complexity and size, structural health monitoring (SHM) systems and safety assessments during construction have become more important topics. As a result, research on these topics is actively being conducted [1,2,3,4,5,6]. SHM and construction safety are the evaluation of the physical responses (displacement, strain, and stress) of civil engineering structures such as bridges, dams, skyscrapers, and aircraft wings, etc. through the use of various types of sensors [7,8]. One of the most important aspects of SHM is the measurement of stress and strain, which are essential for understanding the behavior of structures [9]. Strain is an index indicating a change in an object according to the internal force generated by an external force, and various strain sensor technologies have been developed to measure it accurately. Sensors such as electrical strain gauges (ESG), fiber optic sensors (FOS) [8,10], vibrating wire strain gauges, and fiber Bragg gratings (FBG) [11] have a high degree of accuracy in stress evaluation through strain measurements in the structural field. These sensors are also used for various other purposes, as well as structural fields [12]. However, wire-based attached or embedded sensors used for SHM and construction safety have some drawbacks. Depending on the measurement location and installation method, a great deal of effort is required [13], and there is a risk the sensors could be damaged during installation. In addition, wired-based sensors are sensitive to temperature and humidity, making them difficult to operate based upon weather conditions. Additionally, these kinds of sensors have a short lifespan, which requires a large amount of human and economic cost for maintenance [14]. Nowadays, as structures are becoming taller and larger, the measurement range is increasing, and the installation of wired-based sensors is becoming more difficult and more limited [15]. In order to overcome and improve the chronic limitations of these conventional sensors, research on non-contact remote measurement methods for structural behavior analysis is increasing [16,17].

Recently, a system called terrestrial laser scanning and light imaging detection and ranging (LiDAR), which was first introduced into the geographic information system (GIS) for acquiring geography and terrain, has been expanded in the field of architecture. These systems are used for the development of structural response monitoring systems, such as building information modeling (BIM) and SHM [18,19]. LiDAR system is a new technology that can rapidly acquire three-dimensional (3D) coordinate information using laser scanning and is used for modeling complex and diverse objects [20,21]. In addition, it can generate geometric representations of complex surfaces of objects in real-time with the accurate and dense 3D point cloud, which utilizes numerous points [22,23]. Also, it can remotely acquire 3D coordinate information data for behavior monitoring of buildings, and infrastructure, along with traditional uses such as geography and terrain modeling, geological exploration and surveying, and forest management [24,25,26,27,28]. Unlike conventional wire-based attached or installed sensors, an advantage of a LiDAR system is that it can measure the shape of structures before and after deformation due to the actual external loading [15]. By contrast, conventional sensors can only provide measurements at certain fixed positions or increments. With conventional sensors, there are also other limitations. For example, a structure’s initial shape or large size can make the deformation during manufacture, transportation, and construction difficult to assess using traditional wire-based sensors.

This study describes how to evaluate the strains for the overall shape of a curved thin steel plate under lateral pressure using 3D point cloud data obtained from the LiDAR system. The 9 mm steel plate, used as an inner form for the construction of an outer concrete wall of a liquefied natural gas (LNG) storage tank, was easily deformed by an external force during construction [29,30,31]. The deformation of the plate occurs due to pressure from the fresh concrete during the construction of the outer concrete wall. In order to perform this experiment, only a part of the outer concrete wall of the LNG storage tank was constructed in full-size mock-up, and the ESGs and LiDAR system were used to measure the strain and shape of the steel plate. The 3D point cloud data was then converted to a mesh model through a specific interpolation method, and finally, the strains were calculated by generating a finite element model using the initial shape of the steel plate. The calculated strains were then compared with the measured strains through the actual ESGs to verify the applicability to the strain evaluation method developed in this research.

## 2. Materials and Methods

### 2.1. LiDAR System

LiDAR was developed in the 1960s as a measurement technology for acquiring distance and coordinate information of targets using a laser pulse [32]. It is also possible to record 3D spatial coordinates with many points and coordinate information on the surface of the objects in a relatively short time with a measurement speed of 1000 to 10,000 points or more per second [33,34]. Since the 1970s, it has been used to collect 3D information on GIS. In addition, with the continuous development of laser technology, LiDAR system technologies for various applications have been developed. For example, LiDAR systems are currently used in aircraft, satellites, etc. to precisely observe and analyze the Earth’s atmosphere, geography, and environment. LiDAR is installed on spacecraft and exploration robots as a means to supplement a camera’s performance, and measure the shape and distance of targets. On the ground, simple LiDAR technologies have been commercialized for remote measurement and speed violations, including a 3D scanner for autonomous vehicles. Recently, LiDAR is being used as a core technology for structural behavior monitoring systems in fields such as BIM and SHM, and its utilization and importance are gradually increasing. The advantages of the LiDAR system include the ability to remotely measure inaccessible targets such as coastal areas, which are difficult to assess using ground survey operations, as well as nuclear reactors, areas with complex geography, and high-rise buildings.

**Figure 1:**
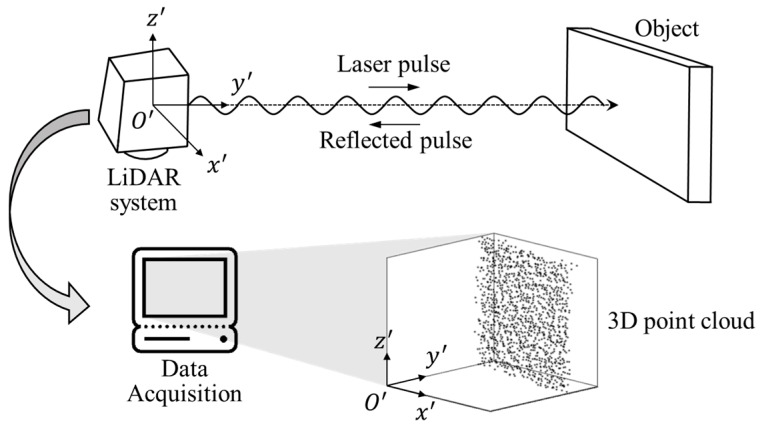
Principle for acquiring 3D coordinate information of the object using the light imaging detection and ranging system.

The 3D laser scanner technology used in structural behavior analysis is based on the time of flight (TOF) principle and uses high energy density and a short period laser pulse signal to obtain 3D coordinate information. The 3D coordinate information is set by calculating the coordinates of the reflection point. This is accomplished by measuring the arrival and return time, velocity, horizontal and vertical angles of the laser pulses reflected by the object, and emitted from the scanner. The measured objects are represented by a point cloud, which consists of several points with coordinate values in 3D space [35]. The coordinate values of each point in the point cloud are generated based on the scanner, which changes according to the position of the scanner. A schematic showing the conceptual composition and principles for obtaining 3D coordinate information using a LiDAR system is shown in Figure 1.

**Figure 2:**
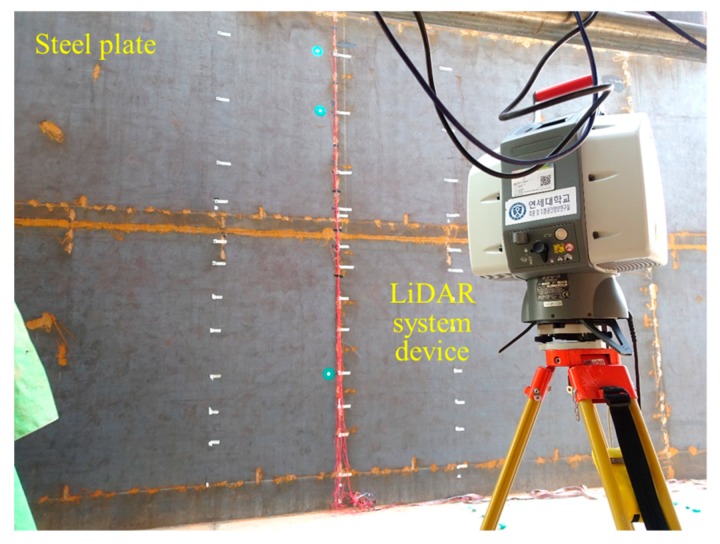
3D LiDAR system device (Leica ScanStation 2).

Unlike contact sensors, a LiDAR system can remotely obtain the 3D coordinate information of entire objects from a single device in a short period of time, allowing engineers to make measurements while maintaining a safe working distance, and without requiring direct access to the objects [36]. LiDAR, therefore, makes it possible to reduce work time and labor resources [37]. In addition, the installation of the LiDAR device and the acquisition process of point cloud data makes it easier to use than wire-based contact sensors. However, during data acquisition, the LiDAR device must be safely installed on the ground where it is secured, and movement around the objects should be minimized so that the emitted laser pulse does not cause interference. In addition, to collect point cloud data using laser pulses emitted and reflected through the LiDAR device, the surface of the test target should not be shiny, so the steel plate was coated in a dark color, as shown in Figure 2. The LiDAR device used in this research is the Leica ScanStation 2, shown in Figure 2, and the specifications and performance are shown in Table 1.

### 2.2. Test Target

Displacement measuring sensors such as linear variable differential transformers and ring-type displacement transducers that measure the displacements of structures can only measure location, without considering the structure’s initial shape. Accordingly, the more complicated and larger the structure, the more limited the understanding of its behavior and deformation shape, making it difficult to predict the overall deformation of the structures from the measured displacements.

**Figure 3:**
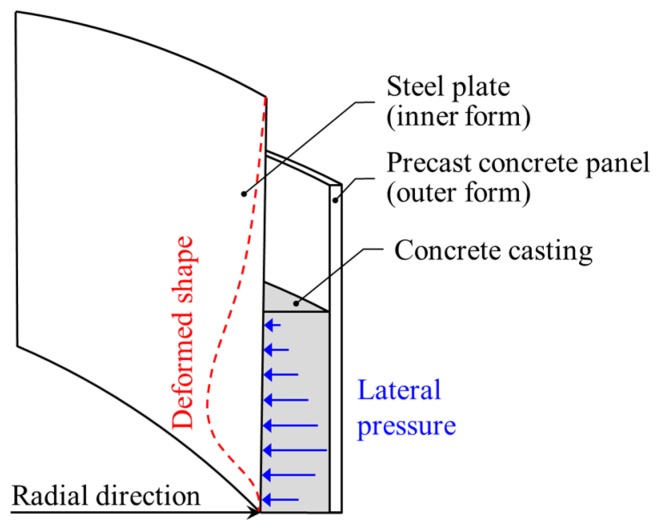
Detailed drawing showing the deformation of the steel plate under lateral pressure during concrete casting.

Although measurements with conventional displacement sensors are limited, we selected a test target that can be measured with the LiDAR system. The chosen test target is a steel plate used for the construction of an outer concrete wall in LNG storage tanks, where it is used as one of the containment structures. The steel plates, used as permanent inner forms, are structures that can be deformed under lateral pressure due to fresh concrete casting, as shown in Figure 3.

**Figure 4:**
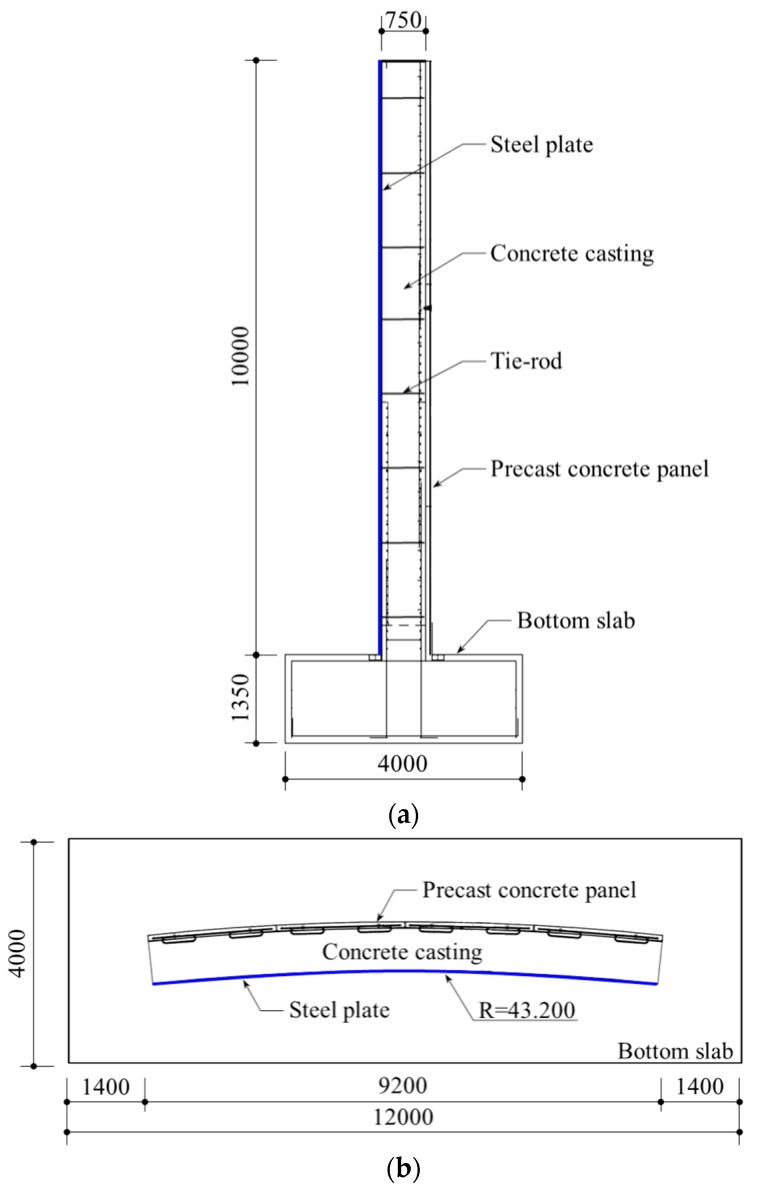
Detailed drawing of the mock-up [Unit: mm]: (**a**) Side view; (**b**) Top view.

**Figure 5:**
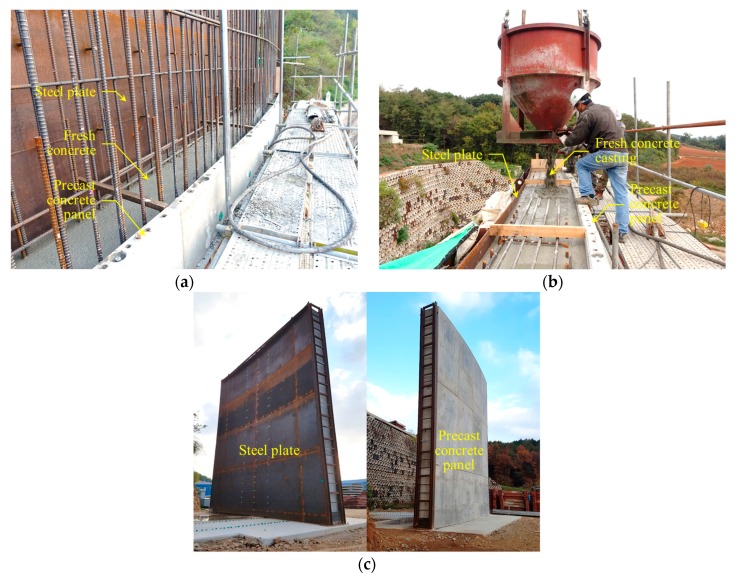
Construction view of the mock-up showing: (**a**) Components of the outer concrete wall; (**b**) Concrete casting between the steel plates and precast concrete panel; (**c**) Completed mock-up.

In summary, as there are few techniques for safely evaluating the construction of outer concrete walls using inner form steel plates, the steel plates were selected as a test target for the LiDAR system. Consequently, a real scale mock-up, which consisted of a partial outer concrete wall, was constructed with the following dimensions: 10 m high, 9.2 m wide, and 0.75 m thick. The steel plate was 9 mm thick, and a detailed drawing of the mock-up is shown in Figure 4. The tie-rods consisted of steel bars, which were used to prevent falling and bulging of the steel plates due to concrete casting. The tie-rods were connected to the steel plates and the precast concrete panels, as shown in Figure 4a. The construction views of the mock-up are shown in Figure 5.

## 3. Methodology for Strain Evaluation

The specific method for evaluating strain is important in the SHM field, as it is based on the 3D coordinate information of the test target acquired from the LiDAR system. The distribution of the point cloud 3D coordinate information of the structure acquired from the LiDAR system may be discretized due to the error of the specifications of the laser scanner. In addition, in order to construct a model suitable for the evaluation of displacements and strains, a numerical model capable of structural analysis must be generated that considers the initial and final deformation shape of the structure, respectively.

The process of the strain evaluation method using the LiDAR system mainly consists of the following steps: (1) acquisition of high-resolution 3D coordinate information data about the initial shape and the deformed shape of the structure from a single LiDAR system device, (2) generation of a point cloud model based on the acquired 3D coordinate information, (3) transformation of the coordinate system of the point cloud model from a LiDAR coordinate system to a structural coordinate system for more accurate structural analysis, (4) conversion of the coordinate-transformed point cloud models to the mesh models using a specific interpolation method, (5) calculation of the relative displacements from the mesh models of initial and deformed shapes, (6) and finally the strains are calculated using a finite element model combined with finite element method (FEM) by inputting the relative displacements in the mesh model of the initial shape.

**Figure 6:**
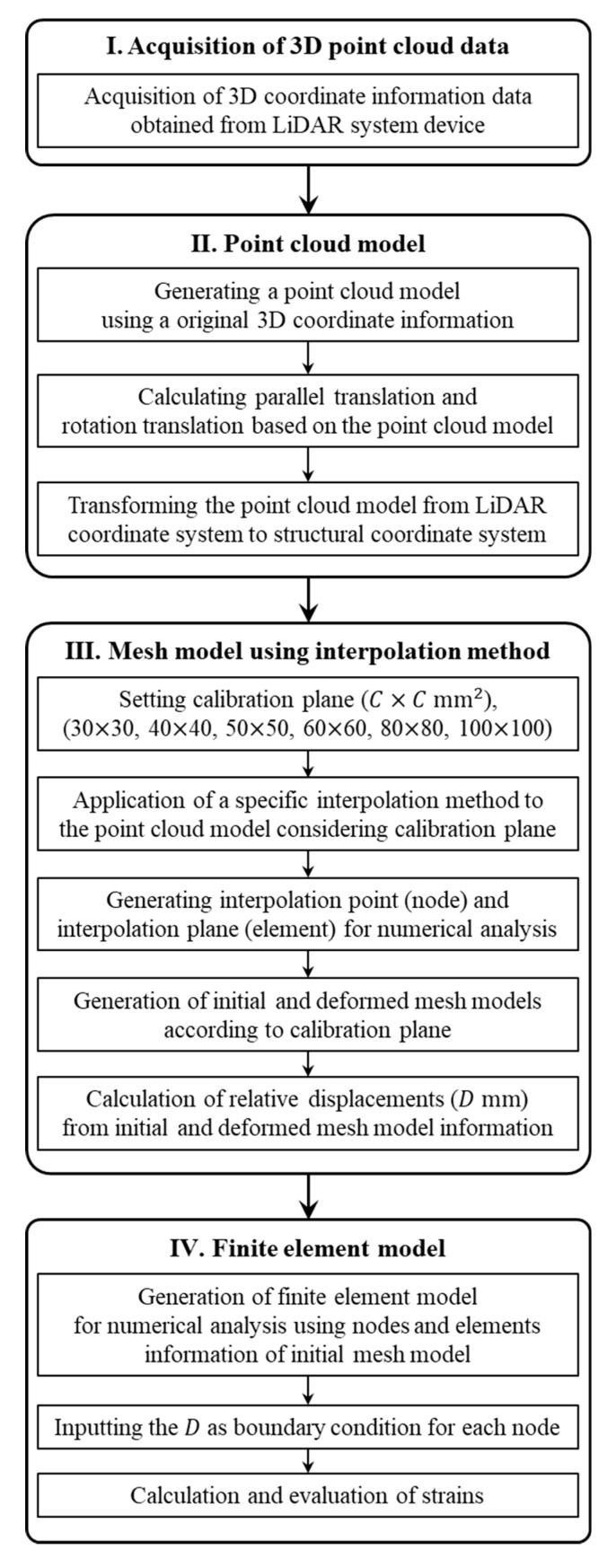
Flowchart for the LiDAR-based strain evaluation method used in this study.

Figure 6 shows the flowchart for the LiDAR-based strain evaluation method proposed in this study.

### 3.1. Acquisition of the 3D Point Cloud Data from LiDAR System

**Figure 7:**
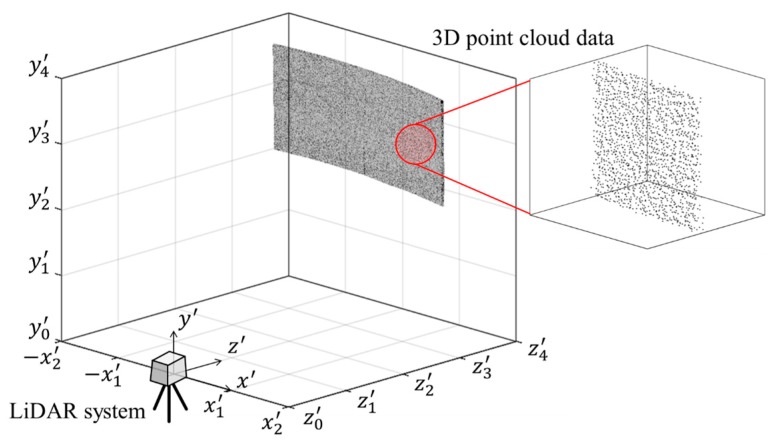
Original point cloud model of the test target.

The 3D point cloud data of the test target acquired through the LiDAR system is generated by setting the LiDAR system device as the reference of the coordinate system. Figure 7 shows a visualization of 3D point cloud data, which includes 3D coordinate information. In this article, this is described as a point cloud model. However, this point cloud model is not suitable for accurately analyzing structural behavior. That is, for accurate structural analysis, a new point cloud model must be generated by transforming the original 3D coordinate information data from the LiDAR coordinate system to the structural coordinate system.

**Figure 8:**
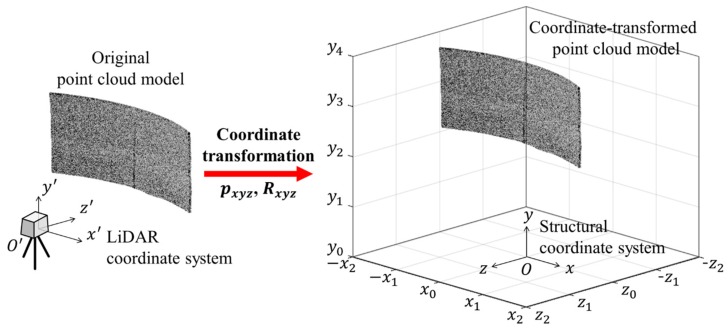
Coordinate transformation of the point cloud model.

In order to perform a coordinate transformation, the origin of the structural coordinate system must be determined to accurately understand the structural behavior by considering the structural deformation and characteristics. In this research, the origin of the structural coordinate system was set at the bottom center of the test target. When the origin position of the coordinate system is set, 3D coordinate transformation, including parallel and rotational translation, is needed, as shown in Figure 8.

The origin of the LiDAR coordinate system is $O^{\prime }\left(x_{o}^{\prime },\;y_{o}^{\prime },\;z_{o}^{\prime }\right)$ and the origin of the structural coordinate system is $O\left(x_{o},\;y_{o},\;z_{o}\right)$, and $x_{o}$, $y_{o}$, and $z_{o}$ are equal to zero. Parallel translation consists of converting the origin from $O^{\prime }\left(x_{o}^{\prime },\;y_{o}^{\prime },\;z_{o}^{\prime }\right)$ of the LiDAR coordinate system to the origin $O\left(x_{o},\;y_{o},\;z_{o}\right)$ of the structural coordinate system, and the parallel translation ($P_{xyz}$) can be represented as follows:

(1) $$P_{xyz}=(x_{o}-x_{o}^{\prime },\;y_{o}-y_{o}^{\prime },\;z_{o}-z_{o}^{\prime })=(-x_{o}^{\prime },\;-y_{o}^{\prime },\;-z_{o}^{\prime })$$

Rotational translation is the transformation of the axis of the LiDAR coordinate system to match the positive direction of the structural coordinate system axis. Rotation angles α, β, and γ are defined according to the positive direction of the axis of the structural coordinate system, and rotational translation ($R_{xyz}$) and $R_{x}$, $R_{y}$, and $R_{z}$ are represented as follows:

(2) $$R_{xyz}=R_{x}R_{y}R_{z}$$

(3) $$R_{x}\left(\alpha \right)=\left[\begin{array}{ccc} 1 & 0 & 0\\ 0 & cos\alpha & -sin\alpha \\ 0 & sin\alpha & cos\alpha \end{array}\right],\;R_{y}\left(\beta \right)=\left[\begin{array}{ccc} cos\beta & 0 & sin\beta \\ 0 & 1 & 0\\ -sin\beta & 0 & cos\beta \end{array}\right],\;R_{z}\left(\gamma \right)=\left[\begin{array}{ccc} cos\gamma & -sin\gamma & 0\\ sin\gamma & cos\gamma & 0\\ 0 & 0 & 1 \end{array}\right]$$

**Figure 9:**
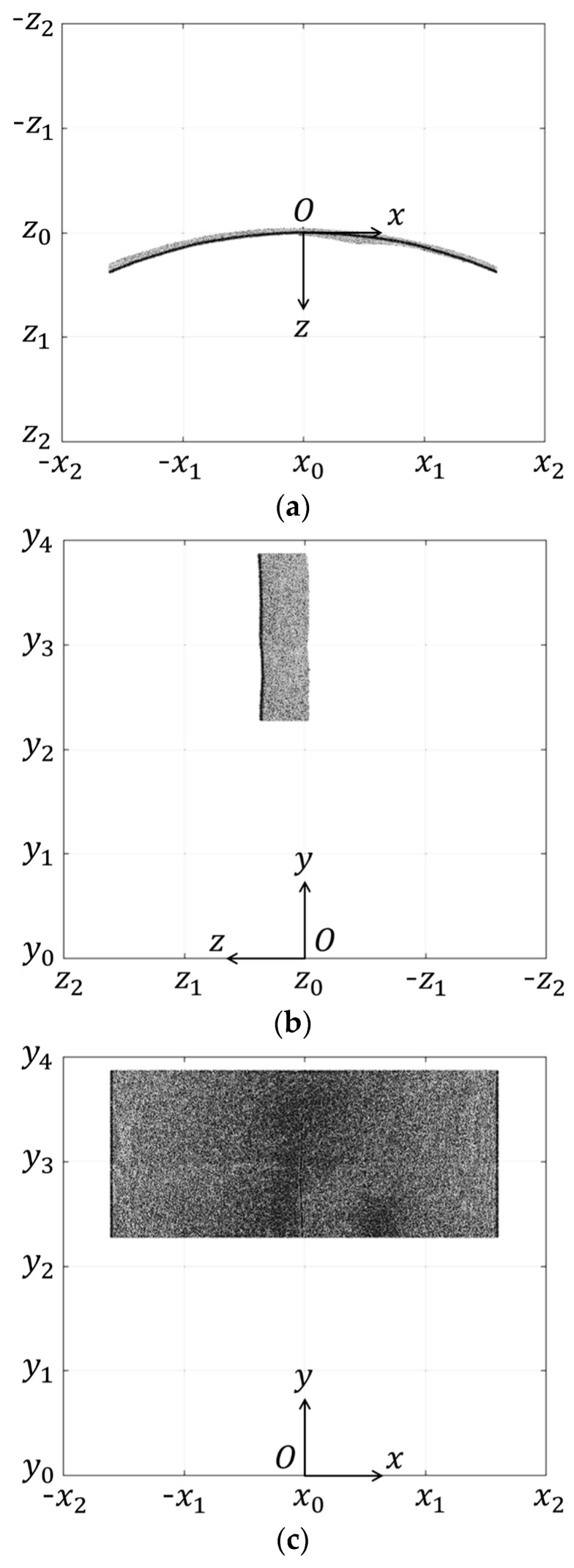
Coordinate-transformed point cloud model in the test target coordinate system: (**a**) Top view; (**b**) Side view; (**c**) Front view.

The 3D point cloud model of the test target transformed from the LiDAR coordinate system to the structural coordinate system through the parallel translation and rotation translation is shown in Figure 9.

### 3.2. Finite Element Model Generation

The coordinate-transformed point cloud models provide visual information about the deformation shape of the structure. However, since the points constituting the point cloud model are irregularly disrupted with noise, the coordinates of some regularly distributed points are needed in order to calculate the displacements at an arbitrary point. Therefore, this section describes the procedures for the mesh models that can accurately identify the 3D coordinate information of the structure based on transformed point cloud models and the generation procedures of the finite element models that can evaluate strain.

**Figure 10:**
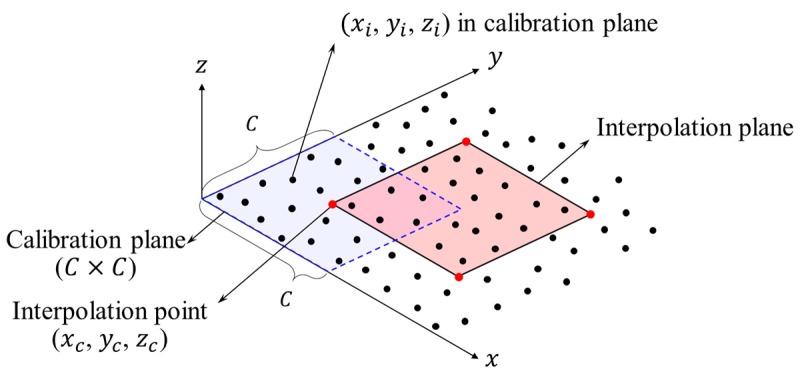
Interpolation method of the coordinate-transformed point cloud model.

First, to generate the mesh models, it is necessary to process data that can be numerically analyzed for discrete point cloud models. To perform this process, a specific interpolation method was used with reference to the digital elevation model (DEM) and the Kriging interpolation method [38,39]. The process of the specific interpolation method used in this research is as follows: (1) according to the number of measurement points, in the transformed point cloud models, M point groups are created by dividing the calibration plane (C×C) at regular intervals, C, in the x and y-xis directions as shown in Figure 10, and M point groups increase as the size of the calibration plane decreases and correspond to the number of measurement points, (2) the first step procedure should be applied to both the transformed point cloud models of initial and deformed shape, (3) finally, the coordinates of the representative points (which is called interpolation point) of each point group ($x_{c}$, $y_{c}$, $z_{c}$), and $x_{c}$ and $y_{c}$ are set to the center points of the calibration plane. Interpolation points and interpolation planes are defined as nodes and elements for generating mesh models. The $z_{c}$ of the interpolation point is calculated as the average of the given cluster points in the calibration plane. The coordinate values of $x_{c}$, $y_{c}$, and $z_{c}$ of the interpolation point are calculated as follows:

(4) $$x_{c}=y_{c}=\frac{C}{2}$$

(5) $$z_{c}=\frac{1}{N}\sum_{i=1}^{N}\left(z_{i}\right)i=\left(1,\;2,\;3,\;\cdots ,\;N\right)$$

where C is the side length of the calibration plane, and the unit is mm. N is the average value of the number of points in the M point groups generated for each calibration plane. When the calibration plane is set to a constant value, N increases as the distance between the LiDAR system device and the structure gets closer, and a dense point cloud data can be obtained.

The mesh models for the initial and deformed shape of the structure are generated by reflecting the size of the calibration plane as a parameter. For obtaining the mesh models suitable for strain evaluation, the appropriate number of interpolation points is required depending on the number of points in the calibration plane. Therefore, the calibration plane was classified into six cases, 30 × 30 ${\mathrm{mm}}^{2}$, 40 × 40 ${\mathrm{mm}}^{2}$, 50 × 50 ${\mathrm{mm}}^{2}$, 60 × 60 ${\mathrm{mm}}^{2}$, 80 × 80 ${\mathrm{mm}}^{2}$, and 100 × 100 ${\mathrm{mm}}^{2}$. Table 2 shows the number of point groups, M, points in the calibration plane, N, interpolation points, and interpolation planes for each case determined by the calibration plane.

**Figure 11:**
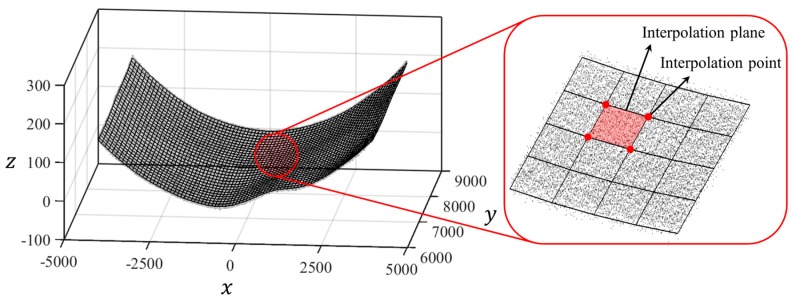
Transformed point cloud and mesh model [Unit: mm].

**Figure 12:**
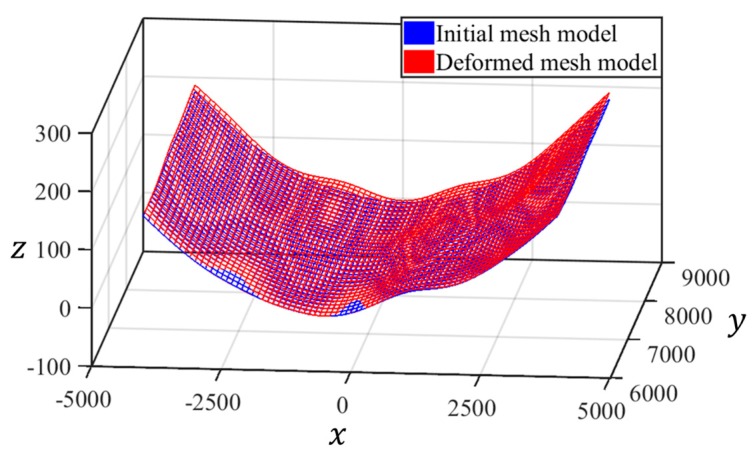
Mesh models of initial and deformed shape [Unit: mm].

The mesh model, which is generated using the specific interpolation method, and the transformed point cloud model, are shown in Figure 11. The mesh models for the initial and deformed shapes are shown in Figure 12.

**Figure 13:**
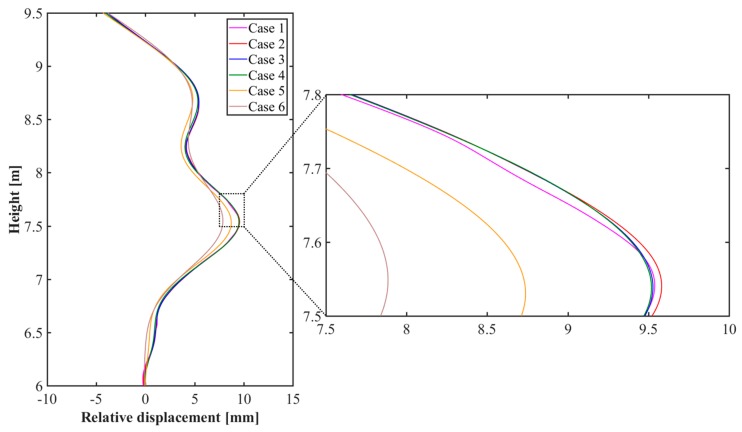
Comparison of the relative displacements for cases 1–6.

The second step is to calculate the relative displacements between the mesh model considering the initial shape, and the mesh model to reflect the deformed shape. The mesh models for the generated initial and deformed shapes have the same number of nodes and elements. In addition, nodes corresponding to each other between the initial mesh model and the deformed mesh model have only the same coordinates of x and y, and z is different. In other words, the difference of z between the corresponding nodes is the relative displacements to be obtained at this step. The relative displacements, D, of each node, can be calculated as follows:

(6) $$D=\sqrt{{\left(x_{c\_i}-x_{c\_d}\right)}^{2}+{\left(y_{c\_d}-y_{c\_i}\right)}^{2}+{\left(z_{c\_d}-z_{c\_i}\right)}^{2}}$$

where ($x_{c\_i}$, $y_{c\_i}$, $z_{c\_i}$) is a coordinate of the node for the initial mesh model, and ($x_{c\_d}$, $y_{c\_d}$, $z_{c\_d}$) is a coordinate of the node for the deformed mesh model. Figure 13 represents the relative displacements calculated from the centerline of the steel plate for each case.

**Figure 14:**
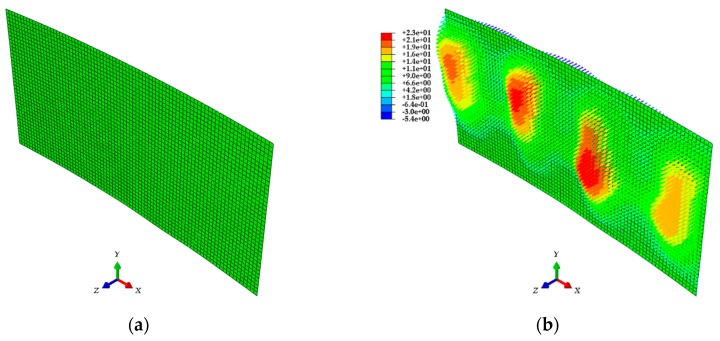
ABAQUS finite element model: (**a**) Initial model; (**b**) Model after inputting the relative displacements as boundary conditions [Unit: mm].

Finally, a finite element model considering the initial shape is composed of shell elements using the commercial finite element analysis (FEA) software ABAQUS [40]. This model is based on the node and element information from the initial mesh data. Among the input parameters for the material properties of this model, the modulus of elasticity is 200 GPa, and the Poisson’s ratio is 0.3. Then, the calculated relative displacements, D, are input into each node as the boundary condition of the test target, and the strain values are calculated using FEA. This method can accurately evaluate the deformation shape based on the discretized point clouds with 3D coordinate information, and load conditions are not required for its application. Figure 14 shows one of the finite element models.

## 4. Strain Evaluation

To verify the applicability of the strain evaluation method using the 3D coordinate information obtained from the LiDAR system, an experimental test was performed on a curved thin steel plate structure subjected to lateral pressure of fresh concrete casting during a simulated construction. For the experiment, ESGs, which are commonly used strain sensors, were attached to the steel plate. The ESGs were continuously used to acquire data during the concrete casting. The LiDAR system was then only applied to capture the initial and deformed shape of the steel plate. The measured values from the ESGs and the strain values were calculated using the finite element models for each case, which was generated from the LiDAR system and compared and verified for applicability.

### 4.1. Test Setup

**Figure 15:**
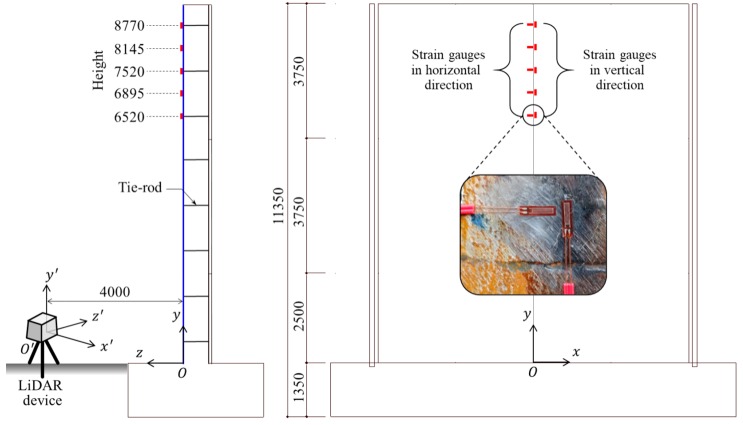
Drawing showing the placement of the electrical strain gauges and LiDAR system relative to the steel plate [Unit: mm].

Steel plates were used as test targets in this research. The plates were chosen in order to demonstrate the application of a 3D coordinate point cloud LiDAR system for the SHM field. The steel plates used in this research were manufactured using the ASTM A516 standard [41], and are typically used as a vapor barrier. After the installation of the steel plates, the ESGs were attached to the exposed surface of the steel plates, as shown in Figure 15. The locations of the ESGs were selected based on the tie-rod positions. The ESGs are in the centerline of the steel plate and attached 6520 mm, 6895 mm, 7520 mm, 8145 mm, and 8770 mm from the ground. In addition, two ESGs were attached at each location in the vertical and horizontal directions. After the attachment of the ESGs, the LiDAR device was installed by adjusting the distance and angle measurement, in order to fully cover the steel plate. Considering this, the LiDAR system device was fixed at a distance of 4 m from the exposed surface of the test target, as shown in Figure 15. Then, only the surface deformation of the test target, except the tie-rods, was measured using the LiDAR system before and after concrete casting.

### 4.2. Results and Comparison

**Figure 16:**
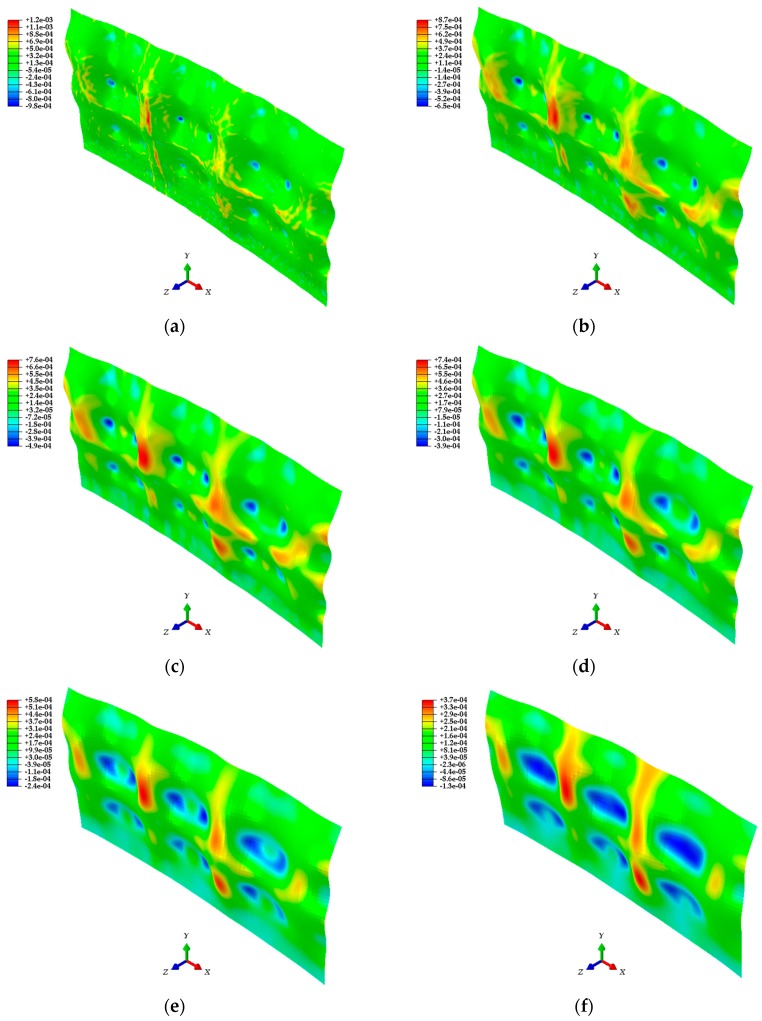
Finite element models showing the strain distribution for: (**a**) Case 1 (30 × 30); (**b**) Case 2 (40 × 40); (**c**) Case 3 (50 × 50); (**d**) Case 4 (60 × 60); (**e**) Case 5 (80 × 80); (**f**) Case 6 (100 × 100).

**Figure 17:**
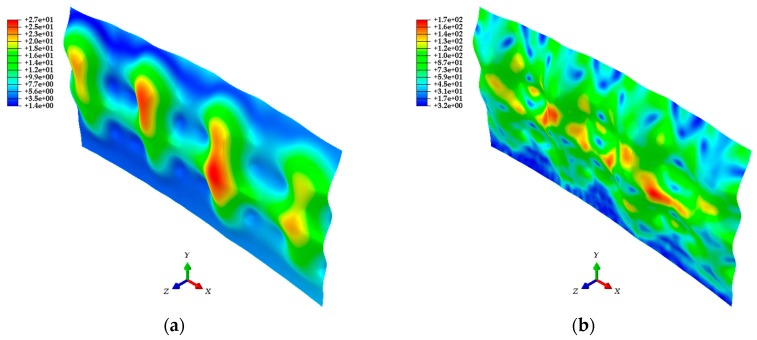
Displacement and stress distribution of the finite element model: (**a**) Distribution of the absolute displacement [Unit: mm]; (**b**) Distribution of the von Mises stress [Unit: MPa].

**Figure 18:**
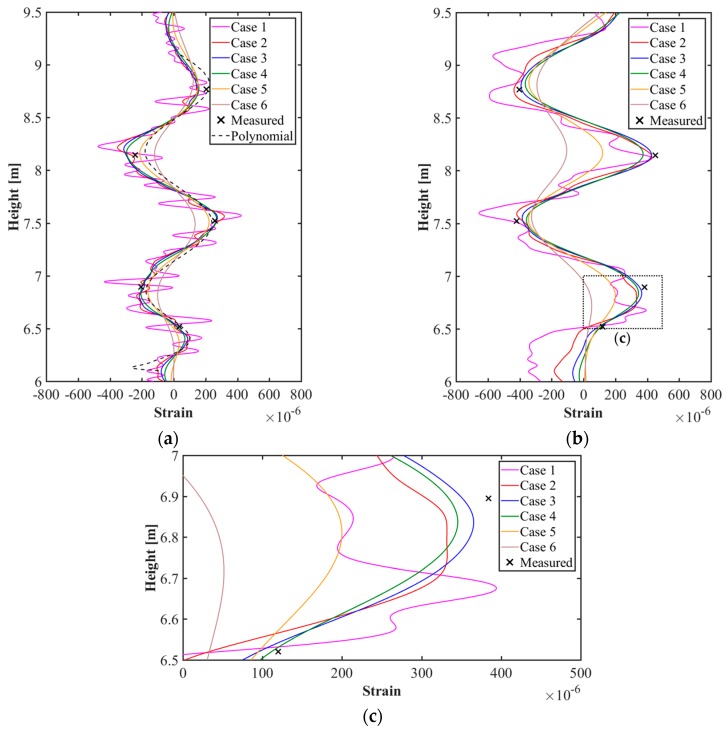
Comparison of strain values from ESGs and finite element models for cases 1–6: (**a**) Vertical strain curves; (**b**) Horizontal strain curves; (**c**) Comparison of detailed strain curves.

In Section 4, point cloud models, mesh models, and finite element models were generated sequentially based on the 3D coordinate information of the test target acquired through the LiDAR system. The ABAQUS software was used for the analysis of finite element models generated with the calibration plane as a parameter. The analysis results shown in Figure 16 give the strain distribution for each finite element model, and consider the initial shape and the deformed shape of the test target. In addition, the distributions of the displacement and stress in the steel plate are shown in Figure 17. Figure 18 shows the comparison of the strain values measured on ESGs attached to the test target and the strain values of each finite element model.

**Figure 19:**
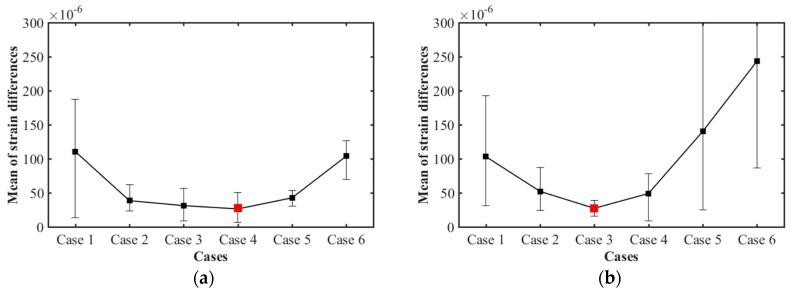
Mean of the strain differences for cases 1–6 between measured strain values of the ESGs, and the calculated values from the finite element models: (**a**) The mean of the strain differences in the vertical direction; and (**b**) Horizontal direction.

Figure 16 confirms that there is a difference in strain distribution, depending on the case. Important factors such as displacement, strain, and stress can be identified at any point on the test target and utilized for understanding the structural behavior. The results are shown in Figure 17. The polynomial strain curve [15], shown in Figure 18a, is a two-dimensional analysis by FEM using a high-order polynomial function, one of the regression methods, based on the point cloud in the center of the steel plate. The reason for the strain curves to fluctuate, as shown in Figure 18 is due to the tie-rods. Since the tie-rods are directly welded to the steel plate, small displacements of the steel plate occur at the tie-rods locations. In other words, as shown in Figure 16 and Figure 17, the convex (expansion) and concave (contraction) phenomena appear on the steel plate depending on the locations of the tie-rods, so the strain curves fluctuate, as shown in Figure 18. The strain curve through the polynomial function shows a similar tendency to the strain curves calculated by the method developed in this study. In addition, the strain curves shown in Figure 18 indicate that strain noise occurs as the calibration plane gets smaller. By comparison, as the calibration plane increases, the noise decreases, but the magnitude of the strain amplitude tends to decrease. The strain values measured from the ESGs are similar to the strain values calculated from the finite element models, but it is not possible to determine which case is more similar to the measured values. Therefore, in order to select a finite element model that includes the most appropriate calibration plane, it is necessary to discriminate based on the mean of the strain differences between the measured values of the ESGs and the calculated values of each finite element model, as shown in Figure 19.

Analysis of the mean strain differences for each case from the measured ESG values showed that the difference in strain decreased and then increased according to the order of the calibration plane. Comparing the mean differences of each case in the vertical direction, the results were: $111\times 10^{-6}$ in case 1, and $39\times 10^{-6}$, $31\times 10^{-6}$, $26\times 10^{-6}$, $43\times 10^{-6}$, and $104\times 10^{-6}$ for cases 2, 3, 4, 5, and 6, respectively. The smallest difference is case 4, with a 60 × 60 calibration plane. In addition, the mean differences in the horizontal direction are $103\times 10^{-6}$ for case 1, and $52\times 10^{-6}$, $27\times 10^{-6}$, $49\times 10^{-6}$, $140\times 10^{-6}$, and $243\times 10^{-6}$ for cases 2, 3, 4, 5 and 6, respectively. In this study, the smallest difference was noticeable in case 3, with a 50 × 50 calibration plane.

## 5. Conclusions

In this study, a method for evaluating the strain at any point on a structure using a 3D coordinate information obtained from the LiDAR system was developed. We demonstrated that the strains could be calculated at any point on the structure based on the validated finite element model. Strain evaluation methods involved several methods: the process of calculating strain through the generation of coordinate-transformed point cloud models, the generation of mesh models using the specific interpolation method with considering the calibration plane, and finally, the generation of finite element models to analyze the structural behavior. The finite element models consisted of six cases with the calibration plane as a parameter. This method was applied to a curved thin steel plate subjected to the lateral pressure caused by fresh concrete. The ESGs were attached to the steel plate to measure the strain during the concrete casting, and at the same time, the initial and deformed shapes of the steel plate were measured by introducing the LiDAR system device. The mesh models of the initial shape were generated from the 3D point cloud data did not have a constant curvature, as in the design drawing, and they are slightly distorted in the vertical and horizontal directions. This kind of distortion and deformation typically occurs during the manufacturing or installation of the steel plate. Analyzing the structural behavior with only the deformed shape without considering these initial deformations can, thus lead to errors and miscalculations. Therefore, it is appropriate to evaluate the structure behavior using a finite element model by measuring both the initial and the deformed shape of the structures using a LiDAR system, and calculating the relative displacements between them.

When comparing the strain values calculated from the finite element models, and considering the initial shape and strain values measured directly from the ESGs, it was confirmed that the differences largely occurred according to the calibration plane. The calibration plane of the cases with the largest error was 30 × 30 (case 1) and 100 × 100 (case 6). In other words, since the shape of the models and the calculated strain values are highly dependent on the calibration plane, it is important to select an appropriate calibration plane to generate an accurate finite element model. The smallest difference between the strain values measured from the ESGs and the strain value calculated from the finite element model was in case 3 in the vertical direction and in case 4 in the horizontal direction.

The most important factor was the number of points, N, in the calibration plane. Points within the calibration plane are denser, as the distance between the LiDAR system device and the target structure is closer, but they become coarser and more sparse as the distance increases. The calibration plane for case 3 was 50 × 50, and the average number of points, N, in the calibration plane to create an interpolation point was 132. In case 4, the calibration plane was 60 × 60, and N was 190. In consideration of this, to create a new calibration plane, as suggested in this article, it is important to consider that the number of points in the calibration plane is about 132–190. However, if the performance of the LiDAR system device is different than the one used in this research, it may be necessary to appropriately increase or decrease the number of points in the calibration plane. In addition, to prevent occlusion when the laser pulses of LiDAR are blocked for complex shapes, it is necessary to determine the measurement positions where no occlusion area occurs and then measure simultaneously using multiple LiDAR devices and overlap all acquired data from each device. In order to measure high-rise buildings, it is advantageous to use high-performance LiDAR devices or unmanned aerial vehicle (UAV) photogrammetry techniques.

By generating a 3D finite element model through this process, it was possible to confirm the strain values for the whole of the structure. Of course, after confirming the locations of the maximum strains occurring in the structure before the experiment, by attaching the ESGs to the determined locations, the structural behavior could be analyzed by the strains measured from the ESGs. However, the more complex structures, the greater the strain can occur at unexpected locations, not just at the points measured by the ESGs. Therefore, measuring a structure using a LiDAR system enables the evaluation of the displacement, strain, and stress distribution in the structure and provides the advantage of evaluating the structural behavior at any point on the structure.

In conclusion, this research demonstrated reliable strain measurements by comparing and verifying the strain values calculated from a LiDAR system and ESGs. Based on the verified finite element model, not only the strains but also structural behavior monitoring behavior such as displacements and stresses can be identified at any point on the structure. Accordingly, the validity and applicability of the developed remote non-contact strain assessment method were established, and this study confirmed that this method could be used for SHM and safety evaluation of complex, high-rise, or inaccessible structures.

## Figures and Tables

**Table 1:** Specifications of the 3D LiDAR device used in this research.

Type	Time of flight pulsed, very-high speed	
Max. measurement distance	300 m	
Max. scanning rate	5000 points/sec	
Field of view	Vertical	270°
	Horizontal	360°
Accuracy of single measurement	Distance	4 mm
	Angle	60 μrad

**Table 2:** The number of point groups, interpolations points, and planes for cases 1–6.

Case	Calibration Plane	*M*	*N*	Interpolation Point	Interpolation Plane
Case 1	30 × 30	35244	47	35244	34846
Case 2	40 × 40	19899	84	19899	19600
Case 3	50 × 50	12880	132	12880	12640
Case 4	60 × 60	8844	190	8844	8645
Case 5	80 × 80	5050	337	5050	4900
Case 6	100 × 100	3240	527	3240	3120
